# Linking the patient experience of foot involvement related to psoriatic arthritis to the International Classification of Functioning, Disability and Health

**DOI:** 10.1093/rap/rkaa028

**Published:** 2020-07-11

**Authors:** Kate Carter, Caterina Tannous, Steven Walmsley, Keith Rome, Deborah E Turner

**Affiliations:** r1 Podiatry Department; r2 Occupational Therapy Department, School of Health and Sciences, Western Sydney University, Sydney, NSW, Australia; r3 Health and Rehabilitation Research Institute, Faculty of Health and Environmental Science, AUT University, Auckland, New Zealand; r4 Podiatry Department, School of Clinical Sciences, Queensland University of Technology, Queensland, Australia

**Keywords:** PsA, foot, International Classification of Functioning, Disability and Health

## Abstract

**Objective:**

The aim was to categorize the patient experience of PsA-related foot involvement by linking it to the International Classification of Functioning, Disability and Health (ICF) framework.

**Methods:**

Concepts, obtained from a previous qualitative investigation of people with PsA and health professionals into their perspective of PsA-related foot involvement, were linked to the full version of the ICF classification. Concepts were linked to the most appropriate ICF category using established linking rules, which enable a systematic and standardized linking process. All concepts were linked independently to the ICF by two investigators, followed by a third investigator for adjudication. The professional backgrounds of the investigators included occupational therapy and podiatry.

**Results:**

More than 100 distinct ICF categories were linked to the interview concepts. The most represented ICF category was body functions (35%), followed by environmental factors (31%), activities and participation (19%) and body structure (15%). Concepts that could not be linked to the ICF were related to coping, aspects of time and knowledge. Health professionals identified a greater proportion of body functions and fewer activity and participation categories compared with patients, indicating a possible mismatch of key concerns. Interdisciplinary group analysis demonstrated merit.

**Conclusion:**

A list of ICF categories was generated, defining aspects of functioning important and relevant to the impact of PsA-related foot involvement. Despite the localized anatomical focus of this study, the effect of foot problems in PsA was linked to all components of the ICF, confirming the profound impact on functioning and daily life.

**Key messages**

- This study comprehensively describes the functioning of people with PsA-related foot involvement and explicitly identifies activities and participation impacted by the condition.
- The list of International Classification of Functioning, Disability and Health categories generated in this study will provide a useful reference to identify what should be included when future foot-specific PsA outcome tools are developed.
- The inclusion of different health disciplines improved the categorization process of the patient experience, highlighting the importance of a multidisciplinary team approach to PsA in future studies.

## Introduction

Previous research has shown merit in linking domains of impact in PsA to the International Classification of Functioning, Disability and Health (ICF) to categorize the effect of global disease [1–3]. Identification of ICF categories relevant to PsA provides a conceptual basis to define what should be measured in the development of outcome measures and assessment tools [1, 2, 4]. As an internationally accepted framework, the ICF has been widely used and advocated by the OMERACT as a reference model to describe better the OMERACT domains relating to functioning and to evaluate health outcome measurement [4, 5]. Previous studies have used the ICF to show that concepts important to people with PsA are not adequately covered by the standard self-report instruments currently used to measure functioning in PsA [6, 7]. The main reasons for this are that existing instruments often contain items that cover different domains (e.g. joints, skin, enthesitis, dactylitis, spine, pain, physical function and quality of life) owing to the heterogeneity of clinical manifestations in PsA, and many of the instruments have been adapted from other rheumatic diseases, with few disease-specific instruments for PsA currently available [8, 9]. Furthermore, incorporation of the patient perspective in the development of outcome measures and domains in PsA is often lacking [10, 11], which limits the potential value of the outcome as discrepancies have been reported between the views of patients and health professionals [12].

Localized pain and disease persistence in the foot in PsA is well recognized [13–17], but limited foot-specific research exists, and there are no outcome measures to provide a comprehensive assessment of foot involvement in PsA and its impact on a person’s function and participation. Previous studies have used the Leeds Foot Impact Scale [18] to assess foot-related disability in PsA [13, 14, 19–21], which consists of sub-scales aligned to the ICF. Whilst this approach has merit in that the Leeds Foot Impact Scale was developed robustly using patient perspectives in RA, it is unlikely to capture the combined musculoskeletal and dermatological impact adequately in PsA. Indeed, few qualitative studies have identified that local foot disease in PsA can cause substantial functional impairments and visual differences, which can negatively impact on emotional well-being and on all aspects of life [22, 23]. To date, little is known about the patient experience of foot involvement and how this might link to the ICF to capture and describe disease impact. The objective of this study was to categorize the patient experience of PsA-related foot involvement by linking descriptive concepts to the ICF.

## Methods

### Concepts from previous qualitative study

**Fig. 1:**
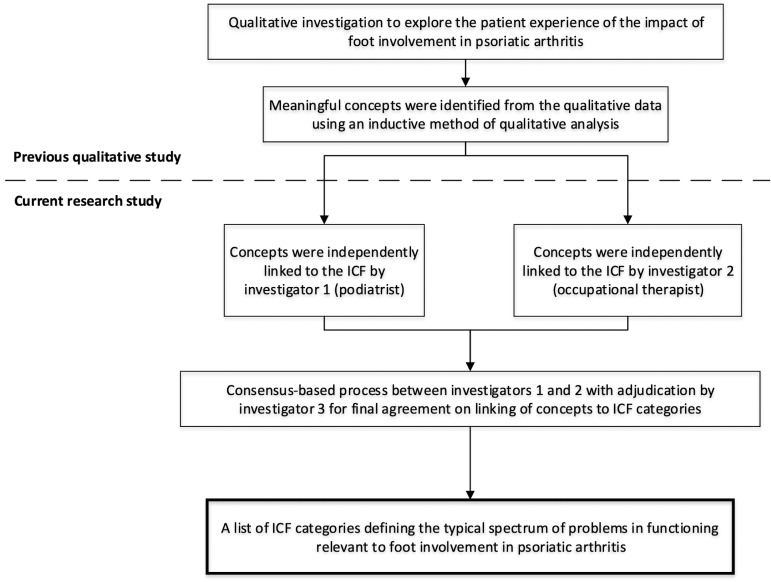
The study design process. ICF: International Classification of Functioning, Disability and Health.

A qualitative study was previously performed based on semi-structured, one-to-one interviews of people with PsA (*n* = 21) and three multidisciplinary focus groups among health professionals [22]. A total of 17 health professionals with clinical experience of managing this patient group participated in three separate focus groups (comprising groups of *n* = 8, *n* = 5 and *n* = 4), including podiatrists, physiotherapists and rheumatologists. The interviews and focus groups covered specific areas of interest, which included foot involvement in PsA, its impact on daily life, experiences with footwear and foot care needs. Each interview and focus group was audio-recorded, transcribed verbatim and analysed using the constant comparative method for qualitative data [24]. This method of thematic analysis combines inductive category coding with a simultaneous comparison of all concepts of meaning obtained from the qualitative data, which are continuously refined, compared and subsequently grouped with similar units of meaning. Meaningful concepts within the text, such as words or sentences containing relevant contextual information, were identified and used in the present study (Fig. 1).

Qualitative research typically uses small sample sizes with a diverse range of participants in order to obtain rich data that allows in-depth exploration and understanding of the research question [25]. A representative sample was sought from the public and private sector, and from lower and higher socioeconomic geographical areas in Australia and New Zealand. Recruitment to the study continued until qualitative data saturation of emerging concepts was achieved [26]. Ethical approval was granted by the South Western Sydney Local Health District (HREC/171/LPOOL/353), the Auckland University of Technology Ethics Committee (AUTEC 17/320) and the Waitemata District Health Board of Auckland New Zealand (RM/3907), and informed consent was obtained from all participants. Detailed descriptions of the preliminary work have been reported previously [22, 23].

### Linking to the International Classification of Functioning, Disability and Health

The structure of the ICF classification is divided into four main components: body structures, body functions, activities and participation, and environmental factors. Each component consists of several chapters, and within each chapter there are second, third and fourth level categories, which are the units of the classification [27]. Within each chapter, the categories are arranged in a stem, branch, leaf scheme. Consequently, a lower-level category shares the attributes of the higher-level category to which it belongs. Hierarchically grouped, the second, third and fourth level categories represent increasingly more detailed frames of reference. Higher levels of linking indicate greater specification. For example, b2 Sensory functions and pain (first level/chapter), b280 Sensation of pain (second level), b2801 Pain in body part (third level) and b28015 Pain in lower limb (fourth level).

Each concept, obtained from the previous qualitative interviews and focus groups, was linked to the most appropriate ICF category according to established linking rules [28] and their updates [29, 30]. The linking rules inform a systematic linking process and facilitate the standardized identification of the linkage between qualitative concepts and ICF categories [28–30]. Using the online ICF classification tool in its full version, each concept was linked to the most precise ICF category. If a concept contained sub-concepts, it was linked to more than one ICF category. For example, a statement from the qualitative data is, ‘Due to the pain in my feet I didn’t want to meet up with my friends’, in which the concepts would be ‘foot pain’ and ‘impact – meeting up with friends’, and the ICF categories linked to them would be ‘b28015 pain in the lower limb’ and ‘d9205 socializing’.

Concepts that could not be linked to the ICF were assigned ‘not covered’ (nc), such as the concept ‘disease progression’ in the present study. Concepts related to personal factors were linked to the ICF whole component ‘personal factors’ (pf), because this ICF component does not include separate categories. Concepts relating to health conditions other than PsA were assigned as ‘health condition’ (hc). If there was insufficient information to make a decision about which ICF category should be linked, it was assigned as not definable (nd), including concepts referring to general health (nd-gh), physical health (nd-ph) and quality of life in general (nd-qol). The ‘other specified’ and ‘unspecified’ categories at the end of each chapter were used if a concept was not explicitly specified.

### Accuracy of analysis

All concepts were independently linked to the ICF by two investigators (K.C. and C.T.). After the independent linking process was complete, the investigators discussed differences in their linking in order to obtain consensus regarding a final set of categories. A third investigator (D.E.T.) assessed all categories, adjudicated cases of disagreement and determined the final category. If required, interview transcripts were reviewed to ensure that the concept had been interpreted accurately. The professional backgrounds of the investigators included occupational therapy and podiatry, and all three investigators undertook self-directed training in linking concepts to the ICF using the eLearning resources developed by the World Health Organization [31]. Full understanding of the concepts and structure of the ICF was required by the investigators before commencing the linking process [30]. In addition, the three investigators had experience of qualitative research methods and 15 years of clinical practice experience.

The degree of agreement between the two investigators in linking concepts to the ICF was calculated using total percentage agreement and the unweighted κ statistic [32]. Data were recorded for each of the four main components of the ICF for linking of the patient and health professional concepts individually and combined. Values of κ can range from zero to one, where one indicates perfect agreement and zero indicates no additional agreement beyond what is expected by chance alone. When interpreting κ statistics, published definitions were used to determine the degree of agreement [33]. The analysis was performed using SPSS v.25 software (SPSS, Inc., Chicago, IL, USA). Descriptive statistics were used to analyse the number and frequency of ICF categories identified.

## Results

A total of 1363 relevant concepts were identified from the interviews of people with PsA and the focus groups with health professionals. The frequency with which each of these concepts was reported among all participants was 4774, of which 924 were from health professionals and 3850 from people with PsA.

### Frequency of ICF categories

One hundred and forty-eight distinct ICF categories were linked to the interview and focus group concepts, which related to body structures (*n* = 17, 12%), body functions (*n* = 48, 32%), activities and participation (*n* = 55, 37%) and environmental factors (*n* = 28, 19%).

The most represented ICF component was body functions (35%), followed by environmental factors (31%), activities and participation (19%) and body structure (15%) (Tables 1–4). The most frequent third level categories were body image (b1801), maintaining one’s health (d5702) and footwear that was linked to general products and technology for personal use in daily living (e1150). The most frequent second level categories were emotional functions (b152), walking (d450), carrying out daily routine (d230) and health services, systems and policies (e580). Also cited frequently was the major life area of remunerative employment (d850), followed by the impact on family (d760) and social life (d920).

**Table 1:** The frequency of International Classification of Functioning, Disability and Health categories for the component body structure that were linked from concepts obtained from people with PsA and health professionals

ICF categories	Generated from health professionals, *n* (%)	Generated from people with PsA, *n* (%)
Body structure (s)		
s75021 Ankle joint and joints foot and toes	73 (33)	305 (34)
s8104 Skin of lower extremity	14 (6)	194 (21)
s8301 Toenails	31 (14)	143 (16)
s7502 Structure of ankle and foot	62 (28)	142 (15)
s75022 Muscles of ankle and foot	19 (9)	53 (6)
s75012 Muscles of lower leg	0	23 (3)
s7302 Structure of hand	4 (2)	13 (1.5)
s750 Structure of lower extremity	0	8 (0.9)
s75023 Ligaments and fasciae of ankle and foot	13 (6)	7 (0.7)
S7703 Extra-articular ligaments, fasciae, extramuscular aponeuroses, retinacula, septa, bursae, unspecified	3 (1.5)	5 (0.5)
s7501 Structure of lower leg	0	4 (0.4)
s75011 Knee joint	0	3 (0.3)
s8300 Fingernails	1 (0.5)	2 (0.2)
s7600 Structure of vertebral column	0	2 (0.2)
s75020 Bones of ankle and foot	0	1 (0.1)
s73021 Joints of hands and fingers	0	1 (0.1)
s7401 Joints of pelvic region	0	1 (0.1)
Total	220	907

ICF: International Classification of Functioning, Disability and Health.

**Table 2:** The frequency of International Classification of Functioning, Disability and Health categories for the component body function that were linked from concepts obtained from people with PsA and health professionals

ICF categories	Generated from health professionals, *n* (%)	Generated from people with PsA, *n* (%)
Body function (b)		
b28015 Pain in lower limb	166 (39.1)	657 (29.5)
b152 Emotional functions	48 (11.3)	245 (11)
b1801 Body image	44 (10.3)	149 (6.7)
b860 Functions of nails	20 (5)	122 (5.5)
b8 Functions of skin	13 (3.1)	119 (5.4)
b126 Temperament and personality functions	9 (2)	114 (5.1)
b770 Gait pattern functions	8 (1.8)	88 (4)
b810 Protective functions of the skin	4 (1)	68 (3)
b7800 Sensation of muscle stiffness	12 (2.8)	64 (2.9)
b2700 Sensitivity to temperature	1 (0.2)	56 (2.5)
b2702 Sensitivity to pressure	0	55 (2.4)
b735 Muscle tone functions	0	45 (2)
b710 Mobility of joint functions	15 (3.5)	43 (1.9)
b760 Control of voluntary movement functions	0	38 (1.7)
b28016 Pain in joints	0	37 (1.6)
b435 Functions of the immune system	37 (9)	34 (1.5)
b7801 Sensation of muscle spasm	0	30 (1.4)
b134 Sleep functions	1 (0.2)	29 (1.3)
b455 Exercise tolerance functions	8 (1.8)	26 (1.2)
b265 Touch functions	0	26 (1.2)
b280 Sensation of pain	0	24 (1.1)
b530 Weight maintenance functions	19 (4.5)	23 (1)
b820 Repair functions of the skin	0	20 (0.9)
b4152 Function of veins	1 (0.2)	16 (0.8)
b1644 Insight	1 (0.2)	15 (0.7)
b1645 Judgement	0	14 (0.6)
b715 Stability of joint functions	0	9 (0.4)
b415 Blood vessel functions	0	9 (0.4)
b5501 Maintenance of body temperature	0	9 (0.4)
b755 Involuntary movement reaction functions	0	6 (0.3)
b4552 Fatigability	2 (0.5)	5 (0.2)
b7303 Power of muscles in lower half of the body	0	5 (0.2)
b6601 Functions related to pregnancy	0	5 (0.2)
b1301 Motivation	2 (0.5)	4 (0.2)
b1642 Time management	0	4 (0.1)
b840 Sensation related to skin	0	4 (0.1)
b270 Sensory function related to temperature and other stimuli (e.g. burning sensation, vibration)	1 (0.2)	2 (0.08)
b1142 Orientation to person	0	2 (0.08)
b830 Other functions of the skin (e.g. sweating)	0	2 (0.08)
b7808 Sensations related to muscles and movement functions, other specified (e.g. strain sensation)	0	2 (0.08)
b730 Muscle power functions	0	2 (0.08)
b7353 Tone of muscles of lower half of body	0	2 (0.08)
b28013 Pain in back	1 (0.2)	1 (0.04)
b1265 Optimism	1 (0.2)	1 (0.04)
b1300 Energy level	1 (0.2)	1 (0.04)
b2800 Generalized pain	5 (1.2)	0
b650 Menstruation functions (e.g. menopause)	2 (0.5)	0
b114 Orientation functions	2 (0.5)	0
Total	424	2232

ICF: International Classification of Functioning, Disability and Health.

**Table 3:** The frequency of International Classification of Functioning, Disability and Health categories for the component activities and participation that were linked from concepts obtained from people with PsA and health professionals

ICF categories	Generated from health professionals, *n* (%)	Generated from people with PsA, *n* (%)
Activities and participation (d)		
d450 Walking	22 (10)	156 (13)
d5702 Maintaining one’s health	21 (9)	146 (12)
d850 Remunerative employment	76 (34)	132 (11)
d5200 Caring for skin	0	57 (5)
d230 Carrying out daily routine	2 (0.9)	57 (5)
d570 Looking after one’s health	4 (1.8)	51 (4.3)
d5204 Caring for toenails	8 (3.6)	46 (3.9)
d760 Family relationships	8 (3.6)	45 (3.8)
d9205 Socializing	8 (3.6)	41 (3.4)
d5700 Ensuring one’s physical comfort	5 (2.2)	41 (3.4)
d920 Recreation and leisure	12 (5.4)	31 (2.6)
d4154 Maintaining a standing position	8 (3.6)	30 (2.5)
d4551 Climbing (e.g. stairs)	2 (0.9)	27 (2.3)
d4153 Maintaining a sitting position	3 (1.3)	25 (2)
d240 Handling stress and other psychological demands	0	22 (1.8)
d4104 Standing	2 (0.9)	21 (1.8)
d9201 Sports	9 (4)	19 (1.7)
d7101 Appreciation in relationships	1 (0.4)	17 (1.4)
d4501 Walking long distances	3 (1.3)	16 (1.3)
d179 Applying knowledge, other specified and unspecified	2 (0.9)	15 (1.3)
d4502 Walking on different surfaces	0	14 (1.2)
d640 Doing housework	0	13 (1.1)
d210 Undertaking a single task	0	13 (1.1)
d6505 Gardening	0	12 (1)
d4602 Moving around outside the home and other buildings	0	12 (1)
d750 Informal social relationships	7 (3)	11 (0.9)
d4552 Running	5 (2.2)	11 (0.9)
d650 Caring for household objects	0	10 (0.8)
d4500 Walking short distances	0	10 (0.8)
d5701 Managing diet and fitness	0	10 (0.8)
d220 Undertaking multiple tasks	0	10 (0.8)
d475 Driving	1 (0.4)	9 (0.7)
d6200 Shopping	0	9 (0.7)
d410 Changing basic body position	4 (1.8)	8 (0.7)
d7600 Parent–child relationships	5 (2.2)	5 (0.4)
d4452 Reaching	3 (1.3)	5 (0.4)
d4103 Sitting	2 (0.9)	5 (0.4)
d5 Self-care	0	5 (0.4)
d4600 Moving around within the home	0	4 (0.3)
d455 Moving around	0	3 (0.25)
d4702 Using public motorized transportation	0	3 (0.25)
d4106 Shifting the body’s centre of gravity	1 (0.4)	2 (0.2)
d9204 Hobbies	0	2 (0.2)
d5402 Putting on footwear	0	2 (0.2)
d5100 Washing body parts	0	2 (0.2)
d740 Formal relationships	1 (0.4)	1 (0.08)
d9100 Informal associations	0	1 (0.08)
d4102 Kneeling	0	1 (0.08)
d5203 Caring for fingernails	0	1 (0.08)
d5403 Taking off footwear	0	1 (0.08)
d4750 Driving human-powered transportation	0	1 (0.08)
d177 Making decisions	0	1 (0.08)
d7201 Terminating relationships	0	1 (0.08)
d4105 Bending	0	1 (0.08)
d4300 Lifting	0	1 (0.08)
Total	225	1195

ICF: International Classification of Functioning, Disability and Health.

**Table 4:** The frequency of International Classification of Functioning, Disability and Health categories for the component environmental factors that were linked from concepts obtained from people with PsA and health professionals

ICF categories	Generated from health professionals, *n* (%)	Generated from people with PsA, *n* (%)
Environmental factors (e)		
e1150 General products and technology for personal use in daily living	150 (29)	712 (40)
e580 Health services, systems and policies	78 (15)	261 (14)
e5800 Health services	44 (8.3)	105 (6)
e1151 Assistive products and technology for personal use in daily living	22 (4)	101 (5.5)
e355 Health professionals	44 (8.3)	89 (5)
e225 Climate	10 (2)	81 (4.5)
e1101 Drugs	32 (6)	80 (4.4)
e445 Attitudes of strangers	5 (1)	64 (3.5)
e310 Immediate family	1 (0.2)	57 (3.2)
e425 Attitudes of colleagues	9 (1.7)	44 (2.4)
e1650 Financial assets	42 (8)	32 (1.7)
e410 Attitudes of immediate family	0	32 (1.7)
e415 Attitudes of extended family	0	32 (1.7)
e2450 Day–night cycles	10 (2)	27 (1.5)
e450 Attitudes of health professionals	15 (3)	22 (1.2)
e420 Attitudes of friends	0	18 (1)
e590 Labour and employment services, system and policies	1 (0.2)	10 (0.6)
e1201 Assistive products and technology for personal indoor and outdoor mobility and transportation	2 (0.4)	9 (0.5)
e340 Personal care providers and personal assistants	0	9 (0.5)
e245 Time-related changes	2 (0.4)	5 (0.3)
e510 Services, systems and policies for the production of consumer goods	1 (0.2)	4 (0.2)
e2250 Temperature	0	4 (0.2)
e115 Products and technology for personal use in daily living	0	4 (0.2)
e515 Architecture and construction services, systems and policies	2 (0.4)	1 (0.03)
e1651 Tangible assets	0	1 (0.02)
e1351 Assistive products and technology for employment	2 (0.4)	1 (0.02)
e5850 Education and training services	44 (8.3)	0
e215 Population	6 (1.2)	0
Total	522	1805

ICF: International Classification of Functioning, Disability and Health.

The majority of concepts reported by people with PsA were linked to third level (more precise) ICF categories across all four components. Environmental factors most relevant to people with PsA were footwear and assistive devices such as insoles, access to health care, support from family and health professionals, drugs and climate. High levels of self-care activity were reported among people with PsA, which covered maintaining and looking after one’s health, and caring for skin and toenails, and these concepts were linked to health-care access, financial assets and assistive devices. Toenail changes, relating to structure and function, were also frequently cited by people with PsA and linked to domains of body image and social relationships. Lack of understanding about the disease was a strong theme from the patient experience and was linked to the attitudes of friends, family, colleagues, strangers and health professionals, but it was difficult to link aspects of knowledge and education that did not fully represent this concept.

About half the number of body function (*n* = 26, 57%) and activities and participation categories (*n* = 27, 49%) were identified by health professionals compared with participants with PsA (*n* = 45, 94% and *n* = 55, 100% respectively), indicating a possible mismatch of key concerns. The majority of concepts reported by health professionals were linked to second level (less precise) ICF categories from the components body functions and activities and participation. More than one-third of the ICF categories identified from the health professional focus group concepts related to environmental factors (*n* = 522, 38%), with the majority being third level ICF categories. This reflected key concerns about the limited access to, and provision of, specialist foot care services, which was the most frequent environmental factor reported by health professionals, followed by concerns relating to footwear restrictions among patients and the lack of training across professionals on the management of rheumatic foot disease.

### Levels of linking

More than half of the ICF categories identified were third level categories (*n* = 76, 51%), followed by second level categories (*n* = 60, 40%). The ICF component that had the most specific categories (higher-level) was body structures, with 44% of concepts being linked to seven fourth level categories (relating to the bones, joints, muscles, ligaments and fascia in the foot and ankle). This was followed by body functions, with 76% of concepts being linked to three fourth level categories, which related mainly to ‘pain in the lower limb’. The two first level categories were self-care (d5) and, more frequently, functions of skin (b8). Psoriatic skin changes were reported mainly in relation to the physical and psychological consequences, which was reflected in the interview concept focusing on the impact of skin change and not specifically on the skin quality (b810) or sensation (b840).

### Difficulty with linking to the ICF

Fifty-seven interview codes containing five concepts could not be linked to the ICF categories, including the disease course (*n* = 27, 47%; which comprised disease variability, duration, progression, chronicity and established foot disease), co-morbidity (*n* = 24, 42%), illness knowledge (*n* = 4, 7%), quality of life in general (such as ‘life is ruined’, ‘life is impossible’ or ‘nightmare’; *n* = 1, 2%) and general physical health (such as ‘debilitating’ or ‘incapacitating’; *n* = 1, 2%). Fifty-nine interview codes containing seven concepts were assigned to the ICF component personal factors, which included coping styles (*n* = 23, 39%), age (*n* = 10, 17%), gender (*n* = 10, 17%), ethnicity (*n* = 5, 8%), family history of inflammatory arthritis (*n* = 9, 15%), lifestyle (*n* = 1, 2%) and concerns and priorities (*n* = 1, 2%) (see Supplementary Tables S1 and S2, available at *Rheumatology Advances in Practice* online).

Concepts that could not be linked precisely to the ICF were related to coping strategies, aspects of time, knowledge of global and local disease, discomfort, rest, swelling, muscle cramp, tendon and enthesis, falls and instability, fatigue and treatment side-effects. Difficulties in linking highly specific information to categories such as sensations of pain, sensations of skin and emotional functions revealed a limitation in the ability of the ICF to discriminate between various effects of the disease (see Supplementary Data S1 and S2, available at *Rheumatology Advances in Practice* online, for a description of the linking process and the concepts that were difficult to link to the ICF).

### Accuracy of analysis

The overall total percentage agreement in the linking of patient and health professional concepts combined ranged from a maximum of 86.3% for body functions to a minimum of 72% for activities and participation. Moderate to very good levels of inter-rater agreement were identified across the ICF components in relation to the linking of patient and health professional concepts combined, ranging from moderate inter-rater reliability for activities and participation at 0.59 (95% CI 0.53, 0.64) to very good for body functions at 0.81 (95% CI 0.78, 0.85) (see Supplementary Data S3, Tables S3 and S4, available at *Rheumatology Advances in Practice* online, for data on the total percentage agreement and Cohen’s κ for inter-rater agreement).

The key difference between the raters was the perception of activity functions (d) and mental and attitudinal functions (b). For example, some aspects of coping were assigned to the ICF category handling stress and other psychological demands (d240) by the first rater, but were assigned to temperament and personality functions (b126) by the second rater. Although coping style is defined as a personal factor present as a pre-morbid state in the linking rules, it could be attributed to a consequent impairment of the disease. This overlap in the meaning of coping resulted in b126 being the sixth most frequently cited body function category and being the most frequent concept assigned to personal factors (*n* = 214, 75%).

## Discussion

To the best of our knowledge, this study is the first to identify ICF categories of importance to people with PsA-related foot involvement and relevant to the health professionals involved in their care. Emergent concepts from the foot-specific qualitative-based work were linked to all components of the ICF, confirming that local disease in the foot in PsA has a broad impact on daily life, with physical, psychological and societal consequences. The OMERACT and the Group for Research and Assessment of Psoriasis and Psoriatic Arthritis (GRAPPA) have endorsed work using the ICF to identify global aspects of functioning in PsA [1, 2]. However, with recognition that hallmark features of PsA are predominant and persistent in the foot and ankle [13–17], the present study describes the extent to which functioning is influenced by body region-specific involvement. Despite the region-specific focus of the present study, the impact of localized disease in the foot was widespread and consistent with previous studies that assessed global PsA disease [1, 2, 6].

Using the ICF, the full spectrum of foot-specific problems in PsA and the dynamic interaction between domains of impact can be understood better. The physical domain of body structure and function was well represented, reflecting the high foot disease burden and unmet need for specialist foot care reported in other studies in PsA [14, 23]. Important concepts in the component body function were body image and emotional well-being. Dermatological problems were frequently linked to both these concepts, indicating that assessment of the consequences of psoriatic skin and toenail involvement could be helpful for effective patient-centred care. Although the impact on daily activity was most pertinent to people with PsA-related foot involvement, environmental and personal factors covered an array of positive and negative aspects that might play an important role in the assessment of functioning. Despite the diverse expression of PsA in the foot, limited research has focused on understanding the impact of local foot disease, especially from the patient perspective [22]. The present study has identified the most typical and relevant aspects of functioning from the patient experience of PsA-related foot involvement using the ICF classification as a universal model and language of functioning. The translation of aspects of functioning into ICF terms enables the meaning of the patient experience to be condensed, defined and compared [4, 34].

Whilst the benefits of early detection and tight control of active foot disease in PsA have been acknowledged [23, 35, 36], recent qualitative research revealed deficiencies in the assessment and management of foot problems related to PsA reported by patients and health professionals in Australia and New Zealand [22, 23]. The need for personalized, targeted assessment and management strategies focused on the manifestations and impact of foot disease in PsA has been identified in previous studies [17, 23, 36]. Establishing the key concerns from the patient perspective is an important step towards identifying ‘what to measure’ in the assessment and management of PsA [2, 4]. The findings from the present study confirm that the views and personal importance attributed to different aspects of functioning vary between and among patients and health professionals, which supports similar published work [11, 37–40]. Linking was to lower-level categories (equating to lower specificity) for concepts obtained from the health professionals, which supports the patient perception of poor understanding of the impact of the disease by health professionals. Experienced health professionals did not appear to appreciate the broader aspects of functional and emotional impact that foot problems have on daily life. Failure to recognize concepts important to people with PsA could negatively influence patient compliance with, and efficacy of, treatment strategies, which suggests a need for more education in this area. Categorization of the effect of PsA-specific foot disease using the ICF framework highlights the value of this approach in the identification of concepts important to both patients and health professionals.

Concepts that could not be linked precisely using the online ICF classification and the shortfalls of the ICF noted in the present study were consistent with those reported in previous work in PsA [1, 2] and in other rheumatic conditions [41–44]. Given that enthesitis is a hallmark feature of PsA, the inadequate representation of the enthesis and tendon by the body structure ICF categories for the foot and ankle significantly reduces specificity in describing localized involvement. Difficulty with linking psychological concepts reflects deficiencies in the ICF and is a major limitation in defining foot disease burden. Gaps and limitations in the linking process should be taken into account in order to reflect functioning accurately, with full conceptual coverage.

Interdisciplinary group analysis demonstrated merit, because differences between the predominantly biomedical approach by podiatry and the biopsychosocial approach by occupational therapy in clinical practice led to additional ICF categories being identified between the health professionals, which related mostly to cognitive functions. The ICF framework adheres to the biopsychosocial model of disease and recognizes that function and health result from a complex interplay of the health components [43]. Occupational therapists have been identified to provide additional valuable perspectives that enhance the application of the ICF as a common framework, which is attributable to the strong conceptual connections between the ICF and occupational therapy models [45].

Despite the lack of evidence on the efficacy of multidisciplinary involvement in the management of people with PsA, this approach has been advocated in published expert reviews [36, 46, 47]. Although it might be considered that occupational therapists do not have a strong traditional role in management of the foot in PsA, the findings of the present study highlight that perspectives from different health professions provide a necessary holistic view on foot functional impairment and its impact that might optimize patient outcomes.

Limitations of the present study include the lack of generalizability, with a sample comprising participants from Australia and New Zealand. Participants in other settings and countries might experience problems with a different frequency or focus. Cross-cultural differences relating to environmental and personal factors might be revealed in other countries. Robust methods to ensure the quality of the linking process were used in this study, including the use of reliability checks, an iterative consensus-based process and multiple raters from different professional backgrounds. However, it remains unclear whether other health professionals would have applied the linking rules differently and decided on different categories, as previously identified [48]. Lastly, this study did not report problems of co-morbidity systematically, and it is difficult to determine their relative contribution to problems in function. Therefore, findings from the present study might be subject to bias, because confounding variables were not adjusted for. However, co-morbidities in PsA are common, and eliminating the impact of co-morbidities comes at the expense of external validity and loss of generalizability in a real-world context.

Future work will be to use the results of the present study and the ICF as a common framework to assess the extent to which existing instruments adequately cover foot-specific concepts in PsA. This definitive list of ICF categories might also be used as a starting point for the development of a new instrument to assess foot-specific functioning for research and in clinical practice, and it provides the opportunity to compare foot-related functioning across other rheumatic diseases [4, 6]. A comprehensive understanding of foot functional impairments, limitations in activities and restriction in societal participation might provide the foundation for the development of targeted assessment and intervention strategies that are mapped to key ICF domains.

Despite the localized anatomical focus of the present study, the effect of foot problems in PsA was linked to all components of the ICF, confirming the profound impact on functioning and daily life. These findings offer new knowledge using the perspectives of patients and health professionals that could inform the development of an instrument to measure the impact of foot involvement in PsA.

## Supplementary Material

rkaa028_Supplementary_Data

## References

[1] Gudu T, Kiltz U, De Wit M, Kvien TK, Gossec L. Mapping the effect of psoriatic arthritis using the International Classification of Functioning, Disability and Health. J Rheumatol 2017;44:193–200. 27980011 10.3899/jrheum.160180

[2] Taylor WJ, Mease PJ, Adebajo A et al Effect of psoriatic arthritis according to the affected categories of the International Classification of Functioning, Disability and Health. J Rheumatol 2010;37:1885–91. 20595273 10.3899/jrheum.091315

[3] Sunkureddi P, Doogan S, Heid J, Benosman S et al Evaluation of self-reported patient experiences: insights from digital patient communities in psoriatic arthritis. J Rheumatol 2018;45:638–47. 29449495 10.3899/jrheum.170500

[4] Boonen A, Stucki G, Maksymowych W et al The OMERACT-ICF reference group: integrating the ICF into the OMERACT process: opportunities and challenges. J Rheumatol 2009;36:2057–60. 19738213 10.3899/jrheum.090357

[5] Stucki G, Boonen A, Tugwell P, Cieza A, Boers M. The World Health Organization International Classification of Functioning, Disability and Health: a conceptual model and interface for the OMERACT process. J Rheumatol 2007;34:600–6. 17343306

[6] Stamm TA, Nell V, Mathis M et al Concepts important to patients with psoriatic arthritis are not adequately covered by standard measures of functioning. Arthritis Rheum 2007;57:487–94. 17394177 10.1002/art.22605

[7] Escorpizo R, Boers M, Stucki G, Boonen A. Examining the similarities and differences of OMERACT core sets using the ICF: first step towards an improved domain specification and development of an item pool to measure functioning and health. J Rheumatol 2011;38:1739–44. 21807795 10.3899/jrheum.110395

[8] Mease PJ. Measures of psoriatic arthritis: tender and swollen joint assessment, Psoriasis Area and Severity Index (PASI), Nail Psoriasis Severity Index (NAPSI), Modified Nail Psoriasis Severity Index (mNAPSI), Mander/Newcastle Enthesitis Index (MEI), Leeds Enthesitis Index (LEI), Spondyloarthritis Research Consortium of Canada (SPARCC), Maastricht Ankylosing Spondylitis Enthesis Score (MASES), Leeds Dactylitis Index (LDI), Patient Global for Psoriatic Arthritis, Dermatology Life Quality Index (DLQI), Psoriatic Arthritis Quality of Life (PsAQOL), Functional Assessment of Chronic Illness Therapy–Fatigue (FACIT‐F), Psoriatic Arthritis Response Criteria (PsARC), Psoriatic Arthritis Joint Activity Index (PsAJAI), Disease Activity in Psoriatic Arthritis (DAPSA), and Composite Psoriatic Disease Activity Index (CPDAI). Arthritis Care Res 2011;63:S64–85. 10.1002/acr.20577 22588772

[9] Gladman DD, Helliwell P, Mease PJ et al Assessment of patients with psoriatic arthritis: a review of currently available measures. Arthritis Rheum 2004;50:24–35. 14730596 10.1002/art.11417

[10] Palominos PE, Gaujoux‐Viala C, Fautrel B, Dougados M, Gossec L. Clinical outcomes in psoriatic arthritis: a systematic literature review. Arthritis Care Res 2012;64:397–406. 10.1002/acr.21552 22147535

[11] Tillett W, Adebajo A, Brooke M et al Patient involvement in outcome measures for psoriatic arthritis. Curr Rheumatol Rep 2014;16:418. 24623563 10.1007/s11926-014-0418-7

[12] Dandorfer SW, Rech J, Manger B, Schett G, Englbrecht M. Differences in the patient's and the physician’s perspective of disease in psoriatic arthritis. Semin Arthritis Rheum 2012;42:32–41. 22424812 10.1016/j.semarthrit.2011.12.003

[13] Turner DE, Hyslop E, Barn R et al Metatarsophalangeal joint pain in psoriatic arthritis: a cross-sectional study. Rheumatology 2014;53:737–40. 24369414 10.1093/rheumatology/ket435 PMC3970567

[14] Hyslop E, McInnes IB, Woodburn J, Turner DE. Foot problems in psoriatic arthritis: high burden and low care provision. Ann Rheum Dis 2010;69:928. 20413569 10.1136/ard.2009.111971

[15] Delle Sedie A, Riente L, Filippucci E et al Ultrasound imaging for the rheumatologist XXXII. Sonographic assessment of the foot in patients with psoriatic arthritis. Clin Exp Rheumatol 2011;29:217–22. 21504659

[16] Galluzzo E, Lischi DM, Taglione E et al Sonographic analysis of the ankle in patients with psoriatic arthritis. Scand J Rheumatol 2000;29:52–55. 10722258 10.1080/030097400750001806

[17] Healy PJ, Groves C, Chandramohan M, Helliwell PS. MRI changes in psoriatic dactylitis—extent of pathology, relationship to tenderness and correlation with clinical indices. Rheumatology 2008;47:92–5. 18077498 10.1093/rheumatology/kem315

[18] Helliwell P, Reay N, Gilworth G et al Development of a foot impact scale for rheumatoid arthritis. Arthritis Rheum 2005;53:418–22. 15934122 10.1002/art.21176

[19] Hyslop E, Woodburn J, McInnes IB et al A reliability study of biomechanical foot function in psoriatic arthritis based on a novel multi-segmented foot model. Gait Posture 2010;32:619–26. 20889342 10.1016/j.gaitpost.2010.09.004

[20] Woodburn J, Hyslop E, Barn R, McInnes IB, Turner DE. Achilles tendon biomechanics in psoriatic arthritis patients with ultrasound proven enthesitis. Scand J Rheumatol 2013;42:299–302. 23286761 10.3109/03009742.2012.747626

[21] Wilkins RA, Siddle HJ, Redmond AC, Helliwell PS. Plantar forefoot pressures in psoriatic arthritis-related dactylitis: an exploratory study. Clin Rheumatol 2016;35:2333–8. 27225246 10.1007/s10067-016-3304-z PMC4989019

[22] Carter K, Walmsley S, Chessman D, Rome K, Turner DE. Perspectives of patients and health professionals on the experience of living with psoriatic arthritis-related foot problems: a qualitative investigation. Clin Rheumatol 2019;38:1605–9. 30617439 10.1007/s10067-018-04411-2

[23] Carter K, Walmsley S, Rome K, Turner DE. Health professional views on the assessment and management of foot problems in people with psoriatic arthritis in Australia and New Zealand: a qualitative investigation. BMC Musculoskelet Disord 2019;20:191. 31054575 10.1186/s12891-019-2572-6 PMC6499957

[24] Morehouse RE, Maykut P. Beginning qualitative research: a philosophical and practical guide. 1st edn. London and New York: Routledge, 1994.

[25] Ritchie J, Lewis J, Elam G. Designing and selecting samples In: Ritchie J, Lewis J, eds. Qualitative research practice. London: Sage, 2009: 77–108.

[26] Francis JJ, Johnston M, Robertson C et al What is an adequate sample size? Operationalising data saturation for theory-based interview studies. Psychol Health 2010;25:1229–1245. 20204937 10.1080/08870440903194015

[27] World Health Organization. International Classification of Functioning, Disability and Health. 2001 http://www.who.int/classifications/icf/en/ and Online ICF Browser http://apps.who.int/classifications/icfbrowser/ (8 February 2020, date last accessed).

[28] Cieza A, Brockow T, Ewert T et al Linking health-status measurements to the International Classification of Functioning, Disability and Health. J Rehabil Med 2002;34:205–10. 12392234 10.1080/165019702760279189

[29] Cieza A, Geyh S, Chatterji S et al ICF linking rules: an update based on lessons learned. J Rehabil Med 2005;37:212–18. 16024476 10.1080/16501970510040263

[30] Cieza A, Fayed N, Bickenbach J, Prodinger B. Refinements of the ICF linking rules to strengthen their potential for establishing comparability of health information. Disabil Rehabil 2019;41:574–83. 26984720 10.3109/09638288.2016.1145258

[31] World Health Organization. ICF eLearning tool. 2015 http://icf.ideaday.de/en/index.html (8 February 2020, date last accessed).

[32] Cohen J. A coefficient of agreement for nominal scales. Educ Psychol Meas 1960;20:37–46.

[33] Landis JR, Koch GG. The measurement of observer agreement for categorical data. Biometrics 1977;33:159–74. 843571

[34] Stamm T, Machold K. The International Classification of Functioning, Disability and Health in practice in rheumatological care and research. Curr Opin Rheumatol 2007;19:184–9. 17278936 10.1097/BOR.0b013e3280148e64

[35] Cresswell L, Chandran V, Farewell VT, Gladman DD. Inflammation in an individual joint predicts damage to that joint in psoriatic arthritis. Ann Rheum Dis 2011;70:305–8. 20980703 10.1136/ard.2010.135087

[36] Patience A, Helliwell PS, Siddle HJ. Focussing on the foot in psoriatic arthritis: pathology and management options. Expert Rev Clin Immunol 2018;14:21–8. 29202587 10.1080/1744666X.2018.1413351

[37] Coenen M, Stamm TA, Cieza A et al Comparing two qualitative methods: individual interviews with focus groups in patients with rheumatoid arthritis. Ann Rheum Dis 2005;64:70. 21706128 10.1007/s11136-011-9943-2

[38] Carr A, Hewlett S, Hughes R et al Rheumatology outcomes: the patient’s perspective. J Rheumatol 2003;30:880–3. 12672221

[39] Hewlett SA. Patients and clinicians have different perspectives on outcomes in arthritis. J Rheumatol 2003;30:877–9. 12672220

[40] Hewlett S, Smith AP, Kirwan JR. Values for function in rheumatoid arthritis: patients, professionals and public. Ann Rheum Dis 2001;60:928–33. 11557648 10.1136/ard.60.10.928 PMC1753375

[41] Campbell A, Hocking C, Taylor WJ. The experience of having psoriasis through the lens of the International Classification of Functioning, Disability and Health (ICF). Australas J Dermatol 2014;55:241–9. 23991891 10.1111/ajd.12103

[42] Stamm TA, Bauernfeind B, Coenen M et al Concepts important to persons with systemic lupus erythematosus and their coverage by standard measures of disease activity and health status. Arthritis Care Res 2007;57:1287–95. 10.1002/art.23013 17907225

[43] Boonen A, van Berkel MO, Cieza A, Stucki G, van der Heijde D. Which aspects of functioning are relevant for patients with ankylosing spondylitis: results of focus group interviews. J Rheumatol 2009;36:2501–11. 19833752 10.3899/jrheum.090156

[44] Stamm TA, Cieza A, Coenen M et al Validating the International Classification of Functioning, Disability and Health comprehensive core set for rheumatoid arthritis from the patient perspective: a qualitative study. Arthritis Rheum 2005;53:431–9. 15934102 10.1002/art.21159

[45] Stamm TA, Cieza A, Machold K, Smolen JS, Stucki G. Exploration of the link between conceptual occupational therapy models and the International Classification of Functioning, Disability and Health. Aust Occup Ther J 2006;53:9–17.

[46] Coates LC, Helliwell PS. Psoriatic arthritis: state of the art review. Clin Med 2017;17:65–70. 10.7861/clinmedicine.17-1-65 PMC6297592 28148584

[47] Taylor WJ. Impact of psoriatic arthritis on the patient: through the lens of the WHO International Classification of Functioning, Health, and Disability. Curr Rheumatol Rep 2012;14:369–74. 22592746 10.1007/s11926-012-0263-5

[48] Coenen M, Cieza A, Stamm TA et al Validation of the International Classification of Functioning, Disability and Health (ICF) Core Set for rheumatoid arthritis from the patient perspective using focus groups. Arthritis Res Ther 2006;8:R84. 16684371 10.1186/ar1956 PMC1779412

