# Supplementary material for: Linking the patient experience of foot involvement related to psoriatic arthritis to the International Classification of Functioning, Disability and Health
Source: Rheumatol Adv Pract. 2020 Jul 11;4(2):rkaa028. doi: 10.1093/rap/rkaa028 (PMC7474858; doi:10.1093/rap/rkaa028)
Supplement: rkaa028_Supplementary_Data [file rkaa028_supplementary_data.docx]

**SUPPLEMENTARY MATERIAL**

**Supplementary Data S1.**

*Examples of linking concepts to ICF categories:*

- “Toe deformity” – s75021: Ankle joint and joints foot and toes*.*
- “Tendon problems - Achilles tendon” - s7502: Structure of ankle and foot.
- “Walking change – Slower” – b770: Gait pattern functions.
- “Daily life impact - Difficulty standing” - d4154: Maintaining a standing position.
- “Footwear choice – Difficulty finding shoes” – e1150: General products and technology for personal use in daily living.

*Examples of more than once concept being linked to an ICF category:*

- Body function ICF category b28015: Pain in lower limb was linked to concepts such as “aching”, “burning”, “deep pain”, “dull”, “generalised pain”, “heavy pain”, “niggling pain”, “nagging pain”, “sharp”, “stabbing”, “stinging”, “sore”, “tender”, “severe”, “shooting” and “throbbing”.
- Body function ICF category b152: Emotional functions was linked to concepts such as “fear”, “embarrassment”, “envy”, “frustration”, “upset”, “distressed”, “worried”, “bad tempered”, “dread” and “depressed”.
- Environmental factor ICF category e1150: General products and technology for personal use in daily living was linked to concepts relating to difficulties with footwear characteristics such as “fit”, “heel-height”, “width”, “closed-in”, “open-toed”, “heel counter” and “fastening”.

*Examples of more than ICF category being linked to a concept that contains sub-concepts:*

- “Family support with toenail cutting” - e310: Immediate family, and d5204: Caring for toenails.
- “Foot pain trigger – Walking” - b28015: Pain in lower limb, and d450: Walking.
- “High cost of footwear” – e1150: General products and technology for personal use in daily living, and e1650: Financial assets.
- “Toenail change – Splits” - s8301: Toenails, and b860: Functions of nails.
- “Work impact - Difficulty sitting for long periods” - d4153: Maintaining a sitting position, and d850: Remunerative employment.
- “Climate/Summer - Difficult wearing closed-in shoes to hide toenails” - s8301: Toenails, b1801: Body image, e225: Climate, and e1150: General products and technology for personal use in daily living.

**Supplementary Table S1. Concepts that were linked to the ICF component personal factors.**

| **ICF categories** | **Generated from health professionals** | **Generated from people with psoriatic arthritis** |
| --- | --- | --- |
|  | **n** | **n** |
| Coping styles | 13 | 201 |
| Family history of inflammatory arthritis | 1 | 19 |
| Age | 10 | 2 |
| Personal concerns and priorities | 6 | 3 |
| Gender-specific | 20 | 0 |
| Ethnicity-specific | 9 | 0 |
| Lifestyle | 3 | 0 |
| **Total** | **62** | **225** |

*ICF* international Classification of Functioning, Disability and Health

**Supplementary Table S2. Concepts that were not covered or were not definable by the ICF classification.**

| **ICF categories** | **Generated from health professionals** | **Generated from people with psoriatic arthritis** |
| --- | --- | --- |
|  | **n** | **n** |
| nc – Illness knowledge | 1 | 41 |
| nc - Health condition (e.g. comorbidities) | 21 | 40 |
| nc - Disease course | 63 | 19 |
| nd-qol (e.g. life ruined) | 1 | 7 |
| nd-ph (e.g. debilitating) | 4 | 5 |
| **Total** | **90** | **112** |

*ICF* international Classification of Functioning, Disability and Health, *nc* not covered, *nd-qol* not definable quality of life, *nd-ph* not definable physical health.

**Supplementary Data S2.**

Body Structures that were not foot-specific were related to spine and hand involvement. Proximal issues at spine level impacted on mobility and stability at foot level, and problems with hands and fingernails were reported by way of comparison with foot and toenail problems.

*Concepts that could not be precisely linked to the ICF.* Swelling was assigned to the ICF category b435: immunological system functions to capture inflammation related to the immune response, which covered concepts such as joint and soft-tissue swelling. Swelling of body parts has previously been linked to b454: water, mineral and electrolyte balance functions [1]. However, the majority of concepts related to generalised, non-specific swelling in the foot, ankle and lower leg that were associated with a variety of factors, which included climate, time of day, activity levels, body position and overall change of foot shape. The swelling was unrelated to inflammation or venous insufficiency and was difficult to code in that respect.

Rest was initially assigned to the ICF category b735: muscle tone functions that refers to the tension present in resting muscles, which was later rejected in a consensus-based discussion among the investigators. Rest is seen as a body function with an activity component and has been linked to b1349: sleep function unspecified and d9209: recreation and leisure unspecified in previous studies [2]. Other categories considered were b4552: fatigability, b455: exercises tolerance functions, b130: energy and drive functions, and pf-coping strategy. However, these categories stem from mental and cardiovascular functions that did not reflect the meaning of rest from the qualitative codes.

Enthesis and tendon disorders were linked to s75022: muscles of ankle and foot as the best-matched ICF category and in order to avoid the non-specific ‘other specified’ category. This hallmark feature of PsA is not fully represented by the structure categories available of the ankle and foot.

It was also noted that once linked to the ICF, the conceptual tie between two or more categories is lost and that the ICF does not explain the relationship between categories. Whilst there are recommendations within the published ICF linking rules [3, 4] for handling this issue, further guidance is required to help preserve relationships among ICF linked information [5].

**Supplementary Data S3.**

*Accuracy of analysis*. In total, 66 additional ICF categories were identified by the 2^nd^ rater during the independent linking process. Nearly half of the additional ICF categories identified were from the component Body Functions (n=31, 47%), with chapter b1: mental function being the most represented including higher-level cognitive functions, temperament and personality, experience of self and time, energy and drive, and orientation functions. Disagreements between the raters occurred most frequently with the 3^rd^ level ICF categories.

The percentage total agreement (PTA) for health professional concepts and the ICF components ranged from a minimum of 63.8% for Activities and Participation to a maximum of 85.5% for Body Functions. Similarly, with the patient concepts the PTA was lowest for Activities and Participation (74.9%), but highest for Body Structures (87.5%). Overall, for patient and health professional concepts combined, the PTA ranged from a maximum of 86.3% for Body Functions to a minimum of 72% for Activities and Participation (Table 7).

Good levels of interrater agreement were identified for the majority of ICF categories in relation to the linking of patient and health professional concepts, ranging from 0.62 (95% confidence interval (CI) 0.55, 0.69) for patient concepts and the Activities and Participation Component to 0.79 (CI 0.72, 0.86) for patient concepts and the Body Structures component. However, very good interrater reliability was identified for Body Functions and the patient concepts at 0.81 (CI 0.78, 0.86), while moderate interrater reliability was established for health professional concepts and Activities and Participation at 0.48 (CI 0.38, 0.58). When patient and health professional concepts were combined, interrater reliability ranged from moderate to very good levels across the ICF components, ranging from moderate interrater reliability for Activities and Participation at 0.59 (CI 0.53, 0.64) to very good for Body Functions at 0.81 (CI 0.78, 0.85) (Table 8).

**Supplementary Table S3. Percentage total agreement between two raters for concepts linked.**

| **ICF component** | **Concepts linked from health professionals (%)** | **Concepts linked from patients (%)** | **Concepts linked from health professionals and patients combined (%)** |
| --- | --- | --- | --- |
| Body Structures | 80.8 | 87.4 | 85.6 |
| Body Functions | 85.5 | 86.5 | 86.3 |
| Activities and Participation | 63.8 | 74.9 | 72.0 |
| Environmental Factors | 82.4 | 85.0 | 84.1 |

*ICF* International Classification of Functioning, Disability and Health

**Supplementary Table S4. Kappa values and 95% CI for interrater agreement between two raters for concepts linked.**

| **ICF component** | **Concepts linked from health professionals (CI)** | **Concepts linked from patients (CI)** | **Concepts linked from health professionals and patients combined (CI)** |
| --- | --- | --- | --- |
| Body Structures | 0.71 (0.61, 0.81) | 0.79 (0.72, 0.86) | 0.76 (0.71, 0.82) |
| Body Functions | 0.70 (0.63, 0.79) | 0.81 (0.78, 0.86) | 0.81 (0.78, 0.85) |
| Activities and Participation | 0.48 (0.38, 0.58) | 0.62 (0.55, 0.69) | 0.59 (0.53, 0.64) |
| Environmental Factors | 0.70 (0.60, 0.80) | 0.72 (0.65, 0.79) | 0.71 (0.65, 0.77) |

*ICF* International Classification of Functioning, Disability and Health, *CI* confidence intervals

**References**

1. Stamm TA, Bauernfeind B, Coenen M, Feierl E, Mathis M, Stucki G, et al. Concepts important to persons with systemic lupus erythematosus and their coverage by standard measures of disease activity and health status. Arthritis Care Res 2007;57:1287-95.
2. Stamm TA, Cieza A, Machold K, Smolen JS, Stucki G. Exploration of the link between conceptual occupational therapy models and the International Classification of Functioning, Disability and Health. Aust Occup Ther J 2006;53:9-17.
3. Cieza A, Brockow T, Ewert T, Amman E, Kollerits B, Chatterji S, et al. Linking health-status measurements to the International Classification of Functioning, Disability and Health. J Rehabil Med 2002;34:205-10.
4. Cieza A, Geyh S, Chatterji S, Kostanjsek N, Üstϋn B, Stucki G. ICF linking rules: an update based on lessons learned. J Rehabil Med 2005;37:212-18.
5. Fayed N, Cieza A, Edmond Bickenbach J. Linking health and health-related information to the ICF: a systematic review of the literature from 2001 to 2008. Disabil Rehabil 2011;33:1941-51.
